# Changes in Macrophage Gene Expression Associated with *Leishmania* (*Viannia*) *braziliensis* Infection

**DOI:** 10.1371/journal.pone.0128934

**Published:** 2015-06-08

**Authors:** Clemencia Ovalle-Bracho, Carlos Franco-Muñoz, Diana Londoño-Barbosa, Daniel Restrepo-Montoya, Carlos Clavijo-Ramírez

**Affiliations:** 1 Centro Dermatológico Federico Lleras Acosta, Bogotá, Colombia; 2 Facultad de Medicina, Universidad Nacional de Colombia, Bogotá, Colombia; 3 Departamento de Biología, Universidad Nacional de Colombia, Bogotá, Colombia; Instituto Oswaldo Cruz, Fiocruz, BRAZIL

## Abstract

Different Leishmania species cause distinct clinical manifestations of the infectious disease leishmaniasis. It is fundamentally important to understand the mechanisms governing the interaction between Leishmania and its host cell. Little is known about this interaction between *Leishmania* (*Viannia*) *braziliensis* and human macrophages. In this study, we aimed to identify differential gene expression between non-infected and *L*. (*V*) *braziliensis*-infected U937-derived macrophages. We deployed a whole human transcriptome microarray analysis using 72 hours post-infection samples and compared those samples with their non-infected counterparts. We found that 218 genes were differentially expressed between infected and non-infected macrophages. A total of 71.6% of these genes were down-regulated in the infected macrophages. Functional enrichment analyses identified the steroid and sterol/cholesterol biosynthetic processes between regulatory networks down-regulated in infected macrophages. RT-qPCR further confirmed this down-regulation in genes belonging to these pathways. These findings contrast with those from studies involving other *Leishmania* species at earlier infection stages, where gene up-regulation for this metabolic pathway has been reported. Sterol biosynthesis could be an important biological process associated with the expression profile of macrophages infected by *L*. (*V*.) *braziliensis*. Differential transcriptional results suggest a negative regulation of the genetic regulatory network involved in cholesterol biosynthesis.

## Introduction

American tegumentary leishmaniasis is a public health problem in Central and South America, affecting 18 countries with approximately 1.5 million new cases each year. Colombia, Brazil and Peru present 75% of the cases of cutaneous leishmaniasis in Latin America [1]. Species within the Viannia subgenus are prevalent in America and are linked to cutaneous and muco-cutaneous leishmaniasis, with *L*. *(V*.*) braziliensis* being the major etiological agent of the latter. The development of this group of diseases is multifactorial, is determined by the infecting species and the host immune system, and depends on the survival and replication of the parasite inside macrophages [2]. Parasite survival depends in part on the capacity of the parasite to counter the leishmanicidal mechanisms of the macrophage and to modulate the host immune response [3]. The parasite has developed different strategies to achieve replication, such as delaying phagosome maturation, inactivating acid hydrolases of the phagolysosome [4, 5], disrupting antigen presentation [6, 7], suppressing the Th1 cell response [8], and altering metabolic pathways in the macrophage [9].

Although many studies have examined macrophage gene expression and the factors that influence leishmaniasis pathogenesis, these studies vary according to the infecting species and the stage of infection. Peacock *et al*. compared the genomes of *L*. *(Leishmania) major*, *L*. *(L*.*) infantum* and *L*. *(V*.*) braziliensis*, revealing 200 differentially distributed genes among the three species. The majority of species-specific genes correspond to *L*. *(V*.*) braziliensis*. The genes differentially distributed between species encode proteins involved in parasite-host interactions and parasite survival in the macrophage [10]. These findings highlight the importance of studying the interaction between macrophages and each individual Leishmania species.

Many of the works examining the effect of Leishmania infection on macrophage gene expression have been conducted using species of the *Leishmania* subgenus [11–14], in contrast with the small number of studies conducted on species of the Viannia subgenus. No studies reporting the global gene expression of macrophages challenged with *L*. *(V*.*) braziliensis* were found. Meanwhile, most studies that have described the gene expression profile of macrophages have been performed during the initial stages of infection, between 0 and 24 hours [12, 13, 15–20]. Few works exist on more advanced stages of infection [8, 11].

The expression profiles of macrophages challenged with different Leishmania species mostly exhibit generalized down-regulation [14, 21]. However, gene expression was up-regulated in macrophages infected with *L*. *(V*.*) panamensis* for 24 hours [22]. In contrast, macrophages infected with *L*. *(L*.*) chagasi* for 24 hours did not show differences between genes with positive regulation and genes with negative regulation [16].

This finding indicates that there are differences in the expression profiles of macrophages depending on both the infecting species and the stage at which the parasite-host interaction is studied, therefore, it could be incorrect to extrapolate the results from studies performed with other species. The objective of this work was to determine the global gene expression profile of macrophages derived from the U937 cell line associated with *L*. *(V*.*) braziliensis* infection and to identify, based on functional enrichment analysis, the biological processes linked to the genes differentially expressed between non-infected macrophages and those infected for 72 hours. Two hundred and eighteen differentially expressed genes were identified in the study, out of which 71.6% exhibited down-regulation. Functional enrichment analyses revealed that sterol biosynthesis, including that of cholesterol, is the most relevant process linked to the expression profile of infected macrophages.

## Methods

### Macrophage differentiation and *in vitro* infection

The U937 cell line *(American Type Culture Collection*—CRL-1593.2, United States) was cultured at an initial concentration of 1X10^5^ cells/mL in a final volume of 5 mL of RPMI-1640 medium (Sigma-Aldrich R410, United States, MO) supplemented with 10% fetal bovine serum (FBS). Cells were incubated at 37°C and 5% CO_2_.

The reference strain of *L*. *(V*.*) braziliensis* (MHOM/BR/00/M2903, Centre National De Reference Des Leishmania, France, Montpellier) was cultured at an initial concentration of 1X10^6^ parasites/mL for six days in Schneider medium (Sigma-Aldrich S9895, United States, MO) supplemented with 10% FBS at 26°C, until the stationary phase was achieved.

The differentiation of 6.75X10^6^ U937 cells into macrophages was induced by treating with 100 ng/mL *phorbol-12-myristate-13 acetate* (PMA) for 120 hours at 37°C and 5% CO_2_ on a glass substrate [23]. The macrophages were infected with *L*. *(V*.*) braziliensis* promastigotes opsonized with inactivated AB^+^ human serum. A ratio of 15 parasites per macrophage was used. Infected macrophages were incubated for two hours at 34°C and then washed three times with PBS (pH 7.4) to remove non-bound parasites. Finally, 10% FBS-supplemented RPMI medium was added, and the samples were incubated at 34°C and 5% CO_2_ for 72 hours. Infection was assessed microscopically by determining the percentage of macrophages that were infected. RNA extraction was performed on experimental units where infection was above 60% [21].

### RNA extraction, cDNA preparation, and microarray hybridization

Two experimental groups were considered for the assay: macrophages infected with *L*. *(V*.*) braziliensis* and non-infected control macrophages. Both groups were processed in the same way, except that RPMI was added to control macrophages instead of parasites. Every infection or control experiment was performed in triplicate.

Total RNA was extracted 72 hours after infection, a stage in which amastigotes were replicating (S1 Fig and S1 Table). Infected and non-infected macrophages were washed three times with sterile PBS (pH 7.4) and treated with 500 μL of *TRIzol Reagent* (Invitrogen 15596–018, United States, CA) following manufacturer protocols. An equivalent volume of 70% ethanol was added to the aqueous phase. This solution was transferred to an *RNeasy Mini Kit* spin column (QIAGEN 74104, Netherlands) to perform RNA purification and precipitation according to the manufacturer’s protocols. RNA quality and quantity were determined using a NanoDrop *2000* spectrophotometer (Thermo Fisher Scientific Inc, United States, MA). The integrity of extracted RNA was evaluated using an *Agilent 2100 Bioanalyzer* system with an *RNA 6000 Nano LabChip* kit (Agilent Technologies Ltd, United States, CA). All extracts had a 260/280 ratio above 1.9 and an RNA integrity number (RIN) greater than 7.8. The extracts were kept at -70°C until they were used.

cDNA preparation and hybridization were performed by ALMAC Diagnostics (Great Britain, Durham) Ribosomal RNA was reduced with a *RiboMinus Human Transcriptome Isolation Kit* (Life Technologies K155001, United States, CA). Single-strand cDNA was synthesized using a *NuGEN Ovation* RNA amplification system (NuGEN Technologies Inc. 3100–12, United States). cDNA was fragmented and labeled with biotin using a *Gene Chip WT Terminal Labeling Kit* (Affymetrix Inc. 900670, United States, CA). Each labeled fragment was hybridized on the *Affymetrix* GeneChip Human Exon 1.0 ST Array (Affymetrix Inc. 900649, United States, CA) for 16 hours at 45°C. Finally, the hybridized arrays were washed, stained and scanned using the *GeneChip Fluidics Station 450* and the *GeneChip Scanner 3000 (Affymetrix Inc*, United States, CA) to obtain raw data of the expression levels.

### Microarray data analysis

The raw data generated by the *Affymetrix Expression Console* (EC) software version 1.1 were pre-processed using the *Robust Multichip Average* (RMA) algorithm based on the standard RMA background correction, quantile normalization, and median polish summarization.

Data quality control employed hierarchical clustering analysis and principal component analysis (PCA). *Exon QC Summary* version 1 was used to evaluate the integrity of the expression data based on *ALMAC Diagnostics Gene Chip Quality Control* standards.

### Identification of differentially expressed genes

Pre-processed data were filtered to eliminate non-informative transcripts based on the intensity of the probe sets versus the background noise (p = 0.01) and the variance (α = 0.8). Analysis was performed with the *Feature Selection Workflow* tool (version102) developed by *ALMAC Diagnostics*.

Differentially expressed transcripts within the experimental groups (*i*.*e*., infected and non-infected macrophages) were identified using analysis of variance (ANOVA) statistical tests and *post-hoc* comparisons. The effect of inflation due to the use of multiple tests was reduced using the false discovery rate (FDR) for the ANOVA p values [24]. Differentially expressed transcripts were selected based on a minimum 1.5 log_2_ fold expression change between groups and p ≤ 0.05. The expression values of the differentially expressed genes were normalized by the probe set median and were clustered hierarchically using *Euclidean distance* and *Ward’s linkage* algorithms.

Raw and processed data were deposited into the *Gene Expression Omnibus* (GEO) database with access number GSE61211 based on MIAME (*Minimum Information About a Microarray Experiment*) guidelines [25].

### Functional enrichment analysis

Two tools were used for this analysis: the *Functional Enrichment Tool* (FET), developed by ALMAC Diagnostics based on Gene Ontology (GO) annotations [26], and the commercial software Ingenuity Pathways Analysis (IPA, Ingenuity Systems, www.ingenuity.com). The analyses enabled the identification and organization of biological entities associated with the lists of differentially expressed genes [27]. Each entity was organized according to the statistical value obtained in the enrichment analysis [28], which was adjusted for “multiple testing” [24]. This analysis was used to evaluate the probability that the association between a specific gene and a biological entity was random.

### Validation of differential gene expression by RT-qPCR

The RNA samples used for microarray analysis were treated with DNAse I (Invitrogen 18068–015, United States, CA) following manufacturer protocols. cDNA used as a template in RT-qPCR was obtained using a *High Capacity RNA-to-cDNA* reverse transcription kit (Life Technologies 4387406, United States, CA). RNA in the absence of reverse transcriptase enzyme was used as a negative control in each reverse transcription experiment.

RT-qPCR was used to validate the results obtained with microarrays. Fourteen genes were selected: 10 were randomly chosen from the group of 218 genes with differential expression, and four were part of the cholesterol biosynthesis pathway. *β2M* and *GNB2L1* were used as housekeeping genes because their expression levels did not vary in infected and non-infected macrophages in microarray assays. The sequences of the primers used to amplify the housekeeping and selected genes are presented in S2 Table.

qPCR assays were performed using a *SsoFast EvaGreen Supermix* kit (Bio-Rad 172–5201, United States, CA) following the manufacturer’s protocols. Assays were run in a CFX96 thermocycler (Bio-Rad, United States, CA).

Expression values were determined through the ΔΔCq method [29] using the Gene Expression module of the software CFX *manager* ^TM^ version 3.0 (Bio-Rad, United States, CA). Non-infected macrophages were used as the control condition to compare the gene expression levels in non-infected and infected macrophages. These assays were repeated three times, with three biological replicates for each of the two experimental conditions.

## Results

### Identification of genes differentially expressed by infected versus non-infected macrophages

Leishmania is capable of modulating the immune response and the signaling pathways of macrophages to promote its survival in the host cell. To determine the effect of *L*. *(V*.*) braziliensis* infection at 72 hours on the gene expression of macrophages derived from the U937 cell line, the expression profiles of non-infected and infected macrophages were compared using microarray analysis.

**Fig 1:**
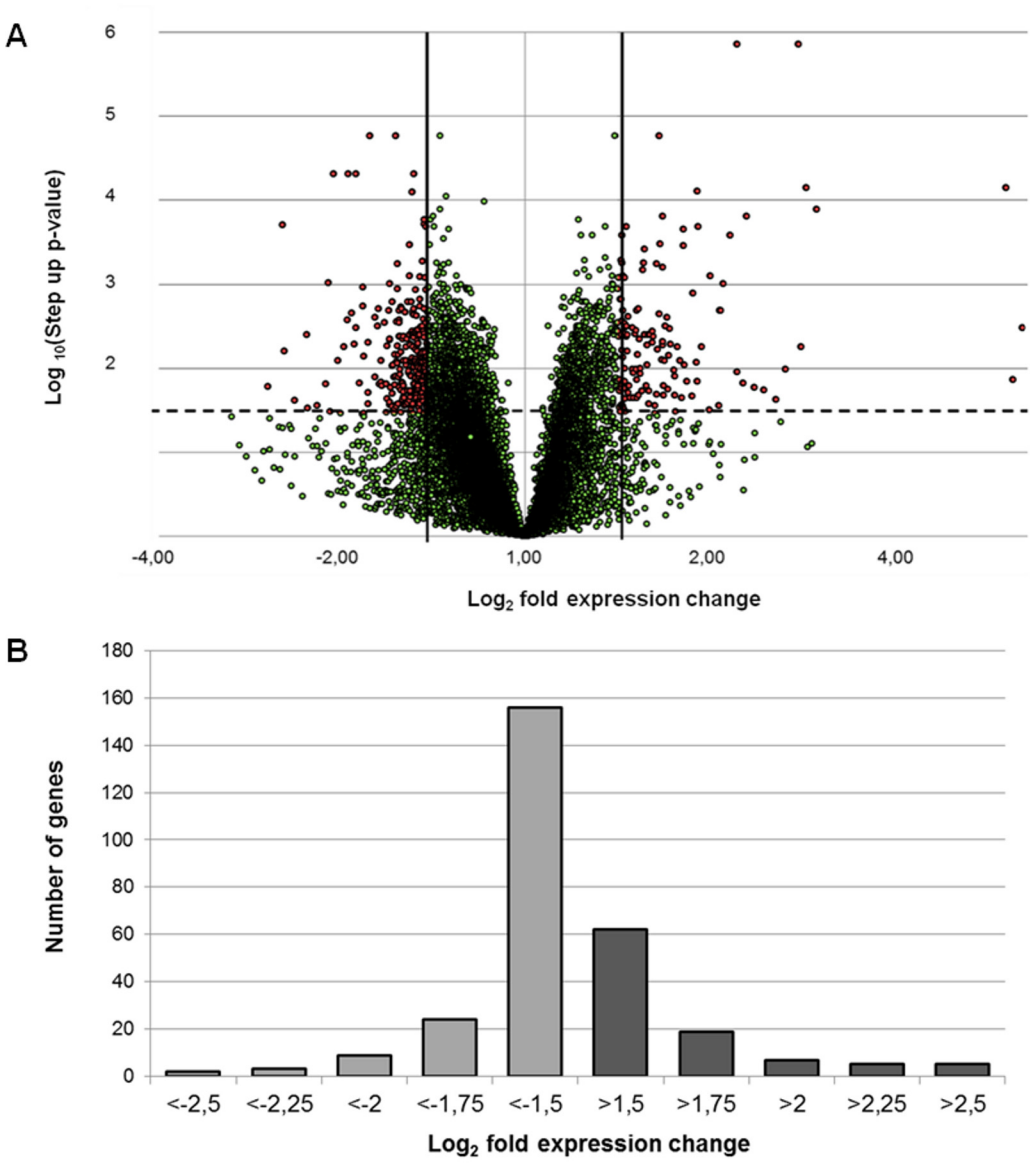
Expression levels of the genes detected through microarray assays. **(a)** Volcano plot of the 12,629 genes detected in the microarray assays. The dashed line marks the limit considered for statistical significance (0.05). The vertical lines indicate the log_2_ fold expression changes of -1.5 and 1.5 used as thresholds to choose the differentially expressed genes. **(b)** Distribution of the fold change values for the 218 differentially expressed genes.

Microarray analyses identified 12,629 genes, out of which 218 were differentially expressed between non-infected macrophages and those infected for 72 hours with *L*. *(V*.*) braziliensis*. The 218 genes were selected based on log_2_ fold expression change greater than 1.5 or less than -1.5, with p < 0.05. From the differentially expressed genes, 71.6% were down-regulated, with log_2_ fold expression change s between -1.5 and -2.63. Over-expressed genes showed log_2_ fold expression change values between 1.5 and 3.01 (Fig 1a and 1b, S3 Table).

Within the group of over-expressed genes, some linked to the macrophage cytoskeleton were detected, such as *TUBA3*, *TUBB2B* AND *SEPT11*. Genes related to the immune response, such as the chemokine *CXCL2* and the gene *WNT7B*, which belongs to the *WNT* family, were also detected. Other genes identified were linked to heavy metal regulation, such as metallothioneins *MT1G* and *MT3*.

Within the group of negatively regulated genes, *HMGCS1*, *STARD5*, *MSMO1* (*SC4MOL*) and *STARD4*, *HMGCR*, *DHCR7*, *SC5DL*, *FADS1*, *FADS2*, *APOB48R* and *APOL6*, which are related to lipid metabolism and transport, stood out. Few of the down-regulated genes were linked to the immune response. These genes included one that encodes interleukin 18 *(IL-18*) and one that encodes the transcription factor signal transducer and activator of transcription 2 (*STAT2*).

**Fig 2:**
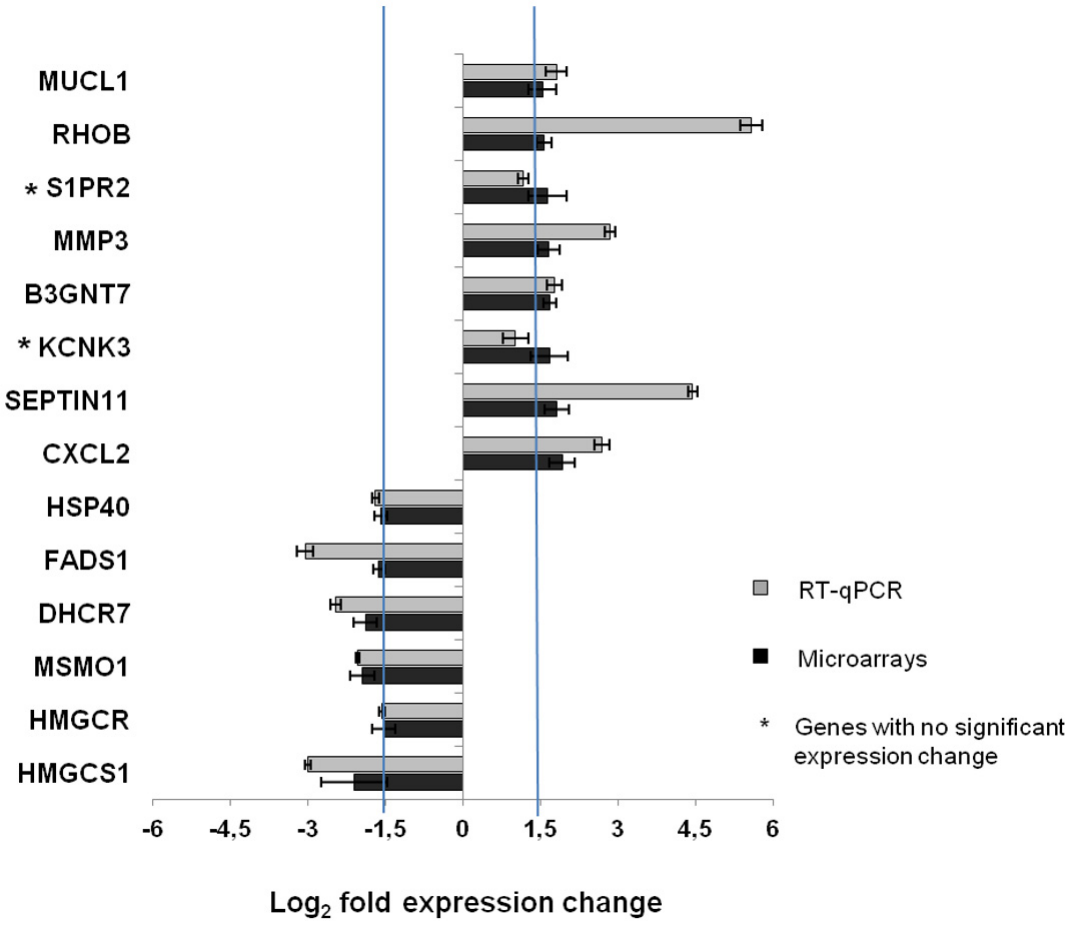
RT-qPCR validation of the expression levels obtained from microarray assays. Out of the 218 genes with differential expression between infected and non-infected macrophages, as identified through microarray assays, 14 genes were evaluated through RT-qPCR to validate their expression levels. Of these genes, 85.7% (12/14) of their RT-qPCR expression data were consistent with the results obtained by microarray analysis.

Fourteen genes were validated through RT-qPCR, and results agreed with those obtained through microarray analysis for 12 genes. However, the genes *potassium channel subfamily K member 3* (*KCNK3*) and *sphingosine-1-phosphate receptor 2* (*S1PR2*) did not exhibit changes in expression through RT-qPCR between infected and non-infected macrophages, while they were identified as over-expressed via microarray analysis (Fig 2).

### Identification of biological entities through functional enrichment analyses

Functional enrichment analysis was performed using the FET tool and the IPA software to identify biological entities linked to the differentially expressed genes.

IPA analysis identified significant differences in mRNA levels of genes encoding proteins involved in steroid biosynthesis, associated with the genes *SC5DL*, *HMGCR* and *DHCR7*, and the degradation of ketone bodies, associated with the genes *HMGCS1* and suggesting this may be an important pathway involved during infection. Analysis using the FET tool identified sterol and cholesterol biosynthesis as the relevant biological processes in the interaction between macrophages and *L*. *(V*.*) braziliensis*, associated with the genes *SC5DL*, *SC4MOL*, *HMGCR*, *C14ORF1*, *HMGCS1* and *DHCR7*.

**Fig 3:**
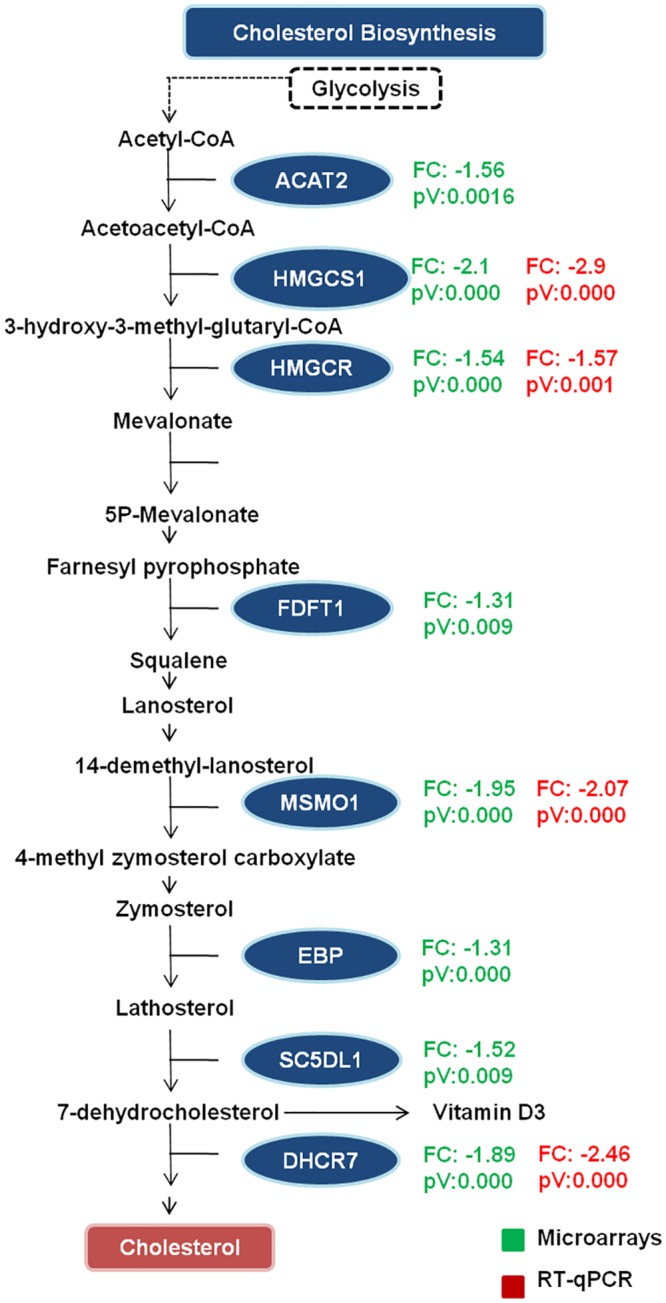
Genes expressed differentially during the infection of macrophages by *L*. *(V*.*) braziliensis* and that participate in the cholesterol biosynthesis pathway. Gene expression results obtained by microarray analysis and confirmed by RT-qPCR are shown.

The results from functional enrichment analysis, along with RT-qPCR validation of expression levels of some of the genes linked to cholesterol biosynthesis, suggest that this biological process could be negatively regulated in macrophages infected with *L*. *(V*.*) braziliensis* for 72 hours (Fig 3).

## Discussion

This study showed that most of the genes differentially expressed between non-infected macrophages and those infected with *L*. *(V*.*) braziliensis* for 72 hours are down-regulated. According to functional enrichment analyses, the biosynthesis of steroids, sterol and cholesterol were among the canonical pathways identified by IPA or FET tools. This study is the first to report the global gene expression of macrophages infected with *L*. *(V*.*) braziliensis* and the associated metabolic pathways.

In the host cell, the macrophage-Leishmania interaction triggers a series of mechanisms aimed at destroying the parasite. However, Leishmania has the capacity to alter the signaling pathways of the macrophage to establish itself in the phagosome for replication [4, 30, 31]. The parasite is capable of inducing alterations in signaling molecules and changes at the transcriptional level through positive or negative macrophage gene regulation. Furthermore, the metabolic processes of the macrophage can change according to the type of parasite [9].

Our results show that *L*. *(V*.*) braziliensis* infection can have a primarily suppressive effect on host gene transcription, as has been previously reported for other Leishmania species [8, 13, 32]. Zhang and peers undertook a meta-analysis in which they included 5 studies on gene expression in macrophages infected by different Leishmania species belonging to the *Leishmania* subgenus, performed at initial infection time points. The authors found that most genes were down-regulated in all of the studies [18]. The general gene repression of macrophages infected by Leishmania, found in these studies, may be related with the capacity of the parasite to alter the expression of genes involved in several pathways causing a cumulative effect of many different down-regulated pathways [11, 33, 34]. These results and those reported in our study differ from those obtained by Ramírez and peers, who reported the over-expression of most of the genes in macrophages infected for 24 hours with *L. (V.) panamensis [22]*. This variability in results suggests the possibility that macrophage gene expression may be linked to the infecting species and the stage of infection in which expression is evaluated.

The expression pattern of macrophage genes upon invasion by a microorganism is dynamic and dependent on the stage of infection. At the initial stages, a pattern of expression of genes linked to the defense mechanisms of the macrophage (the innate and adaptive immune responses) is expected. This phenomenon has been demonstrated in different studies where macrophages have been challenged with *Leishmania spp*.; in the first 24 hours, most of the regulated genes are those related to the immune response [8, 13, 22]. These results contrast with those obtained in this work, where a limited number of genes related to the immune response was identified. Among these, the genes that encode transcription factor *STAT2* and *IL-18* were identified. These two molecules are important for an effective inflammatory response during infection. It is possible that the parasite modulates the expression of these genes to neutralize part of the defense machinery of the macrophage to establish an infection. Other authors have reported similar results [8, 35]. Another gene related to the immune response was chemokine CXCL2, which was over-expressed, a consistent result in studies with different *Leishmania* species (*L*. *(L*.*) amazonensis*, *L*. *(L*.*) mexicana*, *L*. *(L*.*) major* and *L*. *(L*.*) donovani*) [8, 11, 15, 18, 35]. It is possible that the low representation of genes linked to the immune response in this work was due to the stage of macrophage gene expression evaluation, 72 hours post-infection, when the infection has already been established and the macrophage may be adapting its metabolism to confront the infection [9].

To identify the biological processes to which the differentially expressed genes were linked, the microarray results were analyzed using the IPA and FET tools. The results revealed that the pathways with statistical significance were the biosynthesis of steroids, sterol and cholesterol probably related to the process of infection. Although these results were based on the changes registered for few genes important in these pathways, the others genes identified were associated with another biological processes but with low statistical significances.

The findings related to the main canonical pathways suggest at least two scenarios regarding the negative transcriptional regulation of the genes involved in the synthesis of cholesterol in the macrophage. In the first scenario, signaling is normal. That is, the macrophage has high levels of cholesterol and is responding to these levels. In the second scenario, signaling is abnormal. That is, the macrophage has normal (or even below normal) levels of cholesterol, but the signaling pathway is being incorrectly activated, possibly through factors related to the infection.

During the initial interaction between macrophages and promastigotes, cholesterol plays an important role because it is one of the most abundant molecules in lipid rafts, cell membrane micro-domains that serve as platforms for the interaction between different proteins involved in a series of processes including cell signaling, adhesion, invasion, and secretion [36–38]. Previous studies have shown that during the first 24 hours of *L*. *(L*.*) major* infection, there is up-regulation of *HMGCR* at transcriptional level and there is an increase in cytoplasmic lipid droplets, from which cholesterol is an important component. [39]. However, it is important to consider that the regulation of cholesterol biosynthesis exhibits end-product inhibition and that once the cell detects high levels of cholesterol, the molecule itself triggers the negative regulation of its own synthesis. It is possible that this phenomenon is occurring at 72 hours of infection, assuming that cholesterol synthesis increased in the initial moments of infection.

A second scenario that could explain the improper negative regulation of cholesterol can be due to factors related to the parasite. Recently, a mechanism of lipid metabolism regulation related to the protein mammalian/mechanistic target of rapamycin (*mTOR*) has been described. In 2011, Peterson and peers proposed that mTORC1 could regulate the transcription of genes linked to the biosynthesis of cholesterol by modulating the transcription factor sterol regulatory element-binding proteins (*SREBPs*). They propose that *mTORC1* regulates the subcellular localization of *Lipin1*, a transcriptional co-activator and phosphatidic acid phosphohydrolase enzyme, which when dephosphorylated is translocated to the nucleus. The researchers report for NIH3T3 cells, that after pharmacological inhibition with Torin1, *Lipin1* resides in the nucleus, *SREBP*-dependent gene transcription is repressed, and *SREBP* 1 and 2 nuclear levels are reduced [40]. During early infection by *L*. *(L*.*) major*, Jaramillo and peers reported that the glycoprotein gp63 induces mTOR cleavage and inhibits the formation of the *mTORC1* complex [41]. Assuming that this mechanism is shared by *L*. *(V*.*) braziliensis* and that its effects in mTOR signaling prevail at later infection times, it is possible that in the context of our work, gp63 down-regulates *mTOR* reducing *Lipin1* phosphorylation. This in turn, could facilitate the movement of non-phosphorylated *Lipin1* into the nucleus leading to decreased nuclear levels of SREBP-dependent gene transcription.

We did not find any studies in the reviewed literature evaluating the global gene expression of macrophages infected by *L*. *(V*.*) braziliensis* associated to negative regulation of genes related with cholesterol biosynthesis. However, two studies performed on macrophages infected with *L*. *(L*.*) major* reported decreased cholesterol levels 72 hours after infection through the down-regulation of the gene that encodes the enzyme *HMGCR* [42, 43]. In contrast, in two studies performed using macrophages infected with *L*. *(L*.*) amazonensis* and *L*. *(L*.*) major*, the authors observed increases in cholesterol biosynthesis during the initial stages of infection [35, 39]. According to these reports, it can be suggested that cholesterol biosynthesis can be positively or negatively regulated in macrophages infected by *Leishmania spp*. and that this regulation likely depends on the stage of the infection and the infecting species.

To the extent of our knowledge no other study has explored the gene expression pattern of infected macrophages by the parasite *L*. *(V*.*) braziliensis*. However, Novais and peers reported the changes that occur in human skin after infection with *L*. *(V*.*) braziliensis* when compared with normal skin using a genome-wide transcriptional analysis [44]. Those authors reported repression of both cholesterol and free fatty acid biosynthesis in the infected skin samples, findings which are in agreement with our *in vitro* observations in infected U937 derived macrophages with *L*. *(V*.*) braziliensis*.

Our data provide new knowledge about the macrophage—*L*. *(V*.*) braziliensis* interaction at the transcriptional level. The results suggest that some genes related to cholesterol biosynthesis are negatively regulated at 72 hours, the stage of an established infection. Future studies could focus on evaluating the dynamics of cholesterol biosynthesis over time, as the reported results mostly have originated from studies during the initial stages of infection. Furthermore, it would be important to evaluate if the behavior of cholesterol biosynthesis could be dependent on the infecting species.

## Supporting Information

S1 Fig Evaluation of the infection of U937 cell-derived macrophages by *L*. *(V*.*) braziliensis*. **a)** Percent infection and **b)** parasitic load. Macrophages derived from U937 cells were differentiated by incubating 1.2X10^5^ cells for 120 hours with 100ng/mL PMA in RPMI-1640 medium supplemented with 10% FBS at 37°C and 5% CO_2_ on a glass substrate in a 24-well plate. The macrophages were infected with *L*. *(V*.*) braziliensis*-opsonized promastigotes at a 15:1 parasite: macrophage ratio. Percent infection and parasitic load were calculated every 24 hours through Giemsa staining and microscopic determination. The experiment was repeated three times, and each time point was measured in triplicate.
(TIF)

S1 Table Standard error and confidence intervals calculated for parasite load measured at different times after infection of U937 derived macrophages with *L*. *(V*.*) braziliensis* promastigotes. (DOCX)

S2 Table Conditions for amplification of the genes selected to perform RT-qPCR validation of microarray results. Primer sequences and annealing temperatures are reported for each gene.
(DOCX)

S3 Table Differentially expressed genes identified by microarray assays. The 218 genes with differential expression between non-infected macrophages and those infected with *Leishmania braziliensis* are shown. They present log_2_ fold expression greater than 1.5 or less than -1.5, with p < 0.05.
(DOCX)
