# Supplementary figures and images for: Changes in Macrophage Gene Expression Associated with Leishmania (Viannia) braziliensis Infection
Source: PLoS One. 2015 Jun 8;10(6):e0128934. doi: 10.1371/journal.pone.0128934 (PMC4460072; doi:10.1371/journal.pone.0128934)

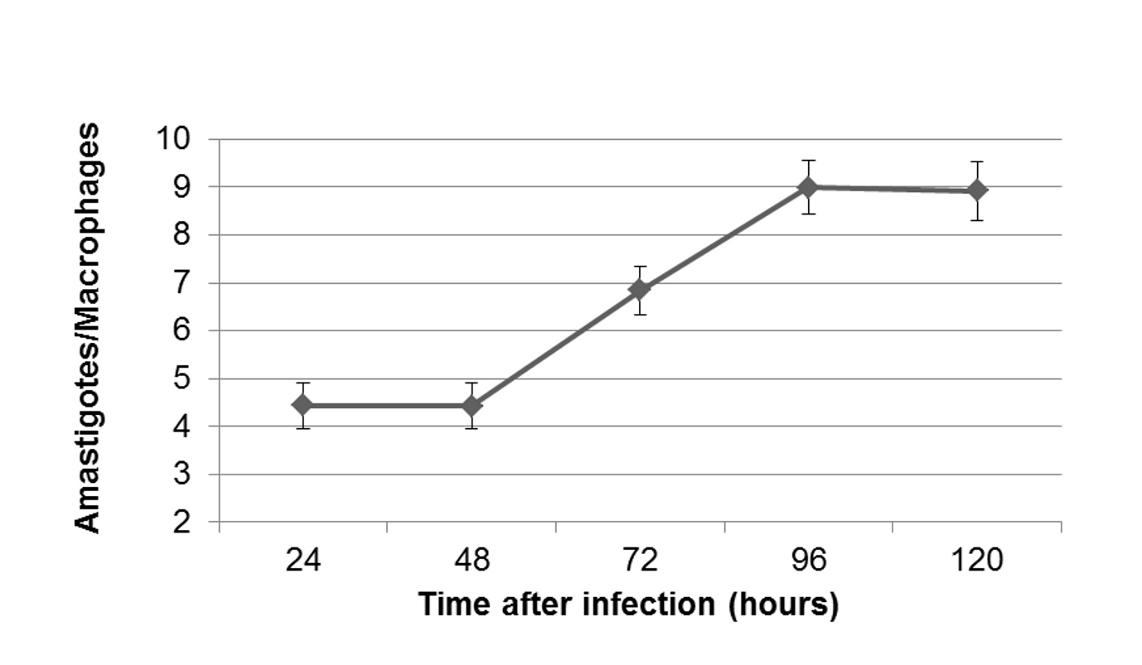

Supplement: S1 Fig — a) Percent infection and b) parasitic load. Macrophages derived from U937 cells were differentiated by incubating 1.2X105 cells for 120 hours with 100ng/mL PMA in RPMI-1640 medium supplemented with 10% FBS at 37°C and 5% CO2 on a glass substrate in a 24-well plate. The macrophages were infected with L. (V.) braziliensis-opsonized promastigotes at a 15:1 parasite: macrophage ratio. Percent infection and parasitic load were calculated every 24 hours through Giemsa staining and microscopic determination. The experiment was repeated three times, and each time point was measured in triplicate. (TIF) [file pone.0128934.s001.tif]
