# Supplementary material for: Changes in Macrophage Gene Expression Associated with Leishmania (Viannia) braziliensis Infection
Source: PLoS One. 2015 Jun 8;10(6):e0128934. doi: 10.1371/journal.pone.0128934 (PMC4460072; doi:10.1371/journal.pone.0128934)
Supplement: S1 Table — (DOCX) [file pone.0128934.s002.docx]

**S2 Table.** Standard error and confidence intervals calculated for parasite load measured at different times after infection of U937 derived macrophages with *L. (V.) braziliensis* promastigotes.

| **Time** | **n** | **Parasite Load mean** | **Standard error** | **95% Confidence Intervals** | |
| --- | --- | --- | --- | --- | --- |
| **24** | 491 | 4,43 | 0,32 | 3,80 | 5,07 |
| **48** | 481 | 4,43 | 0,33 | 3,78 | 5,07 |
| **72** | 472 | 6,84 | 0,41 | 6,04 | 7,65 |
| **96** | 489 | 8,99 | 0,48 | 8,03 | 9,94 |
| **120** | 476 | 8,92 | 0,52 | 7,80 | 9,94 |
