# Supplementary material for: Changes in Macrophage Gene Expression Associated with Leishmania (Viannia) braziliensis Infection
Source: PLoS One. 2015 Jun 8;10(6):e0128934. doi: 10.1371/journal.pone.0128934 (PMC4460072; doi:10.1371/journal.pone.0128934)
Supplement: S2 Table — Primer sequences and annealing temperatures are reported for each gene. (DOCX) [file pone.0128934.s003.docx]

**S3 Table.** Conditions for amplification of the genes selected to perform RT-qPCR validation of microarray results. Primer sequences and annealing temperatures are reported for each gene.

| **Gene** | **Primers sequences** | | **Annealing temperature (˚C)** | |
| --- | --- | --- | --- | --- |
|  |  |  |  |  |
| *GNB2L1* | Fwd | GAGTGTGGCCTTCTCCTCTG | 60.0 ºC |  |
|  | Rev | GCTTGCAGTTAGCCAGGTTC |  |  |
| *B2M* | Fwd | ACTGAATTCACCCCCACTGA | 60.0 ºC | |
|  | Rev | CCTCCATGATGCTGCTTACA |  |  |
| *HMGCS1* | Fwd | GATGTGGGAATTGTTGCCCTT | 62.0 ºC | |
|  | Rev | ATTGTCTCTGTTCCAACTTCCAG |  |  |
| *HMGCR* | Fwd | AGCTTGCCCGAATTGTGTGT | 63.0 ºC | |
|  | Rev | CCAATGCCCATGTTCCAGTT |  |  |
| *MSMO1* | Fwd | GCCAGAGACATGGGAAAACC | 62.0 ºC | |
|  | Rev | GCACAACCAAAGCATCTTGC |  |  |
| *DHCR7* | Fwd | CTCCTGCAGGGGTTGTGAAC | 63.0 ºC | |
|  | Rev | CGAAGGTGGAGACGGCATAG |  |  |
| *B3GNT7* | Fwd | CTGGGACGTGACCACCACTA | 62.0 ºC | |
|  | Rev | GACCGACTTGACAACCACCA |  |  |
| *CXCL2* | Fwd | TGCTGAAAAATGGCAAATCC | 61.0 ºC | |
|  | Rev | CATTAGGCGCAATCCAGGT |  |  |
| *FADS1* | Fwd | CTTTGCCACCTTTTGGGTGT | 63.0 ºC | |
|  | Rev | TGTCCCTTTGTGGCCATGTA |  |  |
| *HSP40* | Fwd | AGGCAGCGGACAAAGAGGTAAA | 63.0 ºC | |
|  | Rev | CTGGGTCCTCTCTGCTGCTT |  |  |
| *KCNK3* | Fwd | ACCAAAGCTGCGGAGAGAAG | 63.0 ºC | |
|  | Rev | GAGTGAGGGGGAAGCAACAC |  |  |
| *MMP3* | Fwd | TGAGGACACCAGCATGAACC | 63.0 ºC | |
|  | Rev | GCATCACCTCCAGAGTGTCG |  |  |
| *MUCL1* | Fwd | CCAGCTACTGGTCCTGCTGA | 63.0 ºC | |
|  | Rev | CCCAACCCATTTGGGTAAAA |  |  |
| *RHOB* | Fwd | AGAGAGCTAGGCCGAGTCCA | 63.0 ºC | |
|  | Rev | GCGTTCTCTCGCTGCGCTT |  |  |
| *SEPTIN11* | Fwd | AGGGAATAACCGCGAATGCT | 63.0 ºC | |
|  | Rev | CTGCTGAGGCGGTGAGAAGT |  |  |
| *S1PR2* | Fwd | GGGCAGGAGCACTTTACCAC | 63.0 ºC | |
|  | Rev | GAGAACGACATGAACCCAGGA |  |  |
