# Supplementary material for: Changes in Macrophage Gene Expression Associated with Leishmania (Viannia) braziliensis Infection
Source: PLoS One. 2015 Jun 8;10(6):e0128934. doi: 10.1371/journal.pone.0128934 (PMC4460072; doi:10.1371/journal.pone.0128934)
Supplement: S3 Table — The 218 genes with differential expression between non-infected macrophages and those infected with Leishmania braziliensis are shown. They present log2 fold expression greater than 1.5 or less than -1.5, with p < 0.05. (DOCX) [file pone.0128934.s004.docx]

**S4 Table. Differentially expressed genes identified by microarray assays.** The 218 genes with differential expression between non-infected macrophages and those infected with *Leishmania braziliensis* are shown. They present fold changes greater than 1.5 or less than -1.5, with p < 0.05.

| **Symbol** | **Assignment** | **Transcript ID** | **Fold-Change** | **p-value** |
| --- | --- | --- | --- | --- |
| *ACTR1A* | ARP1 actin-related protein 1 homolog A, centractin alpha (yeast) | 3304406 | -1,50 | 0,0002 |
| *ALDH18A1* | aldehyde dehydrogenase 18 family, member A1 | 3301512 | -1,80 | 0,0000 |
| *ALPP /// ALPPL2* | alkaline phosphatase, placental-like 2 | 2532294 | 1,52 | 0,0000 |
| *ANAPC16* | anaphase promoting complex subunit 16 | 3251353 | -1,68 | 0,0002 |
| *APOB48R* | apolipoprotein B48 receptor | 3686635 | -2,28 | 0,0001 |
| *APOBEC3C* | apolipoprotein B mRNA editing enzyme, catalytic polypeptide-like 3C | 3945572 | -1,51 | 0,0081 |
| *APOL6* | apolipoprotein L, 6 | 3944243 | -1,50 | 0,0017 |
| *AQP9* | aquaporin 9 | 3595594 | -1,50 | 0,0016 |
| *ATP8B4* | ATPase, class I, type 8B, member 4 | 3623552 | -1,84 | 0,0000 |
| *B3GNT7* | UDP-GlcNAc:betaGal beta-1,3-N-acetylglucosaminyltransferase 7 | 2531908 | 1,68 | 0,0001 |
| *BAHCC1* | BAH domain and coiled-coil containing 1 | 3737874 | -1,52 | 0,0074 |
| *BCL7B* | B-cell CLL/lymphoma 7B | 3056108 | -1,53 | 0,0004 |
| *BMP8B /// OXCT2 /// BMP8A* | bone morphogenetic protein 8a // bone morphogenetic protein 8b | 2331511 | 2,10 | 0,0000 |
| *C12orf4* | chromosome 12 open reading frame 4 | 3441215 | -1,51 | 0,0095 |
| *C12orf47* | chromosome 12 open reading frame 47 | 3471753 | -1,52 | 0,0001 |
| *C12orf59* | chromosome 12 open reading frame 59 | 3404626 | -1,50 | 0,0090 |
| *C14orf1* | chromosome 14 open reading frame 1 | 3572461 | -2,50 | 0,0000 |
| *C14orf138* | chromosome 14 open reading frame 138 | 3563687 | -1,61 | 0,0009 |
| *C15orf51* | dynamin 1 pseudogene | 3641597 | 1,56 | 0,0019 |
| *C17orf49* | chromosome 17 open reading frame 49 | 3708201 | -1,61 | 0,0036 |
| *C19orf61* | chromosome 19 open reading frame 61 | 3864597 | -1,53 | 0,0001 |
| *C1QL2* | complement component 1, q subcomponent-like 2 | 2573016 | 1,50 | 0,0006 |
| *C20orf111* | chromosome 20 open reading frame 111 | 3906821 | -1,52 | 0,0000 |
| *C3orf37* | chromosome 3 open reading frame 37 | 2641577 | -1,52 | 0,0000 |
| *C4orf38* | chromosome 4 open reading frame 38 | 2795904 | 1,52 | 0,0006 |
| *C9orf123* | chromosome 9 open reading frame 123 | 3198289 | -1,59 | 0,0012 |
| *C9orf153 /// ISCA1* | iron-sulfur cluster assembly 1 homolog (S. cerevisiae)// chromosome 9 open reading frame 153 | 3212919 | -1,60 | 0,0097 |
| *C9orf41* | chromosome 9 open reading frame 41 | 3210130 | -1,59 | 0,0029 |
| *C9orf70 /// GLIS3* | chromosome 9 open reading frame 70 | 3160500 | 1,53 | 0,0010 |
| *C9orf85* | chromosome 9 open reading frame 85 | 3174429 | -1,52 | 0,0056 |
| *CA5BP /// CA5B* | carbonic anhydrase VB, mitochondrial | 3969855 | -1,51 | 0,0000 |
| *CARM1 /// YIPF2* | Yip1 domain family, member 2 // coactivator-associated arginine methyltransferase 1 | 3850576 | -1,55 | 0,0019 |
| *CCNB1IP1* | cyclin B1 interacting protein 1 | 3555300 | -1,52 | 0,0013 |
| *CCNB2* | cyclin B2 | 3595979 | -1,90 | 0,0002 |
| *CCNT1* | cyclin T1 | 3453218 | -1,52 | 0,0014 |
| *CDKN1B* | cyclin-dependent kinase inhibitor 1B (p27, Kip1) | 3405440 | -1,61 | 0,0000 |
| *CHMP4A /// MDP1* | chromatin modifying protein 4A// magnesium-dependent phosphatase 1 | 3557947 | -1,67 | 0,0000 |
| *CHRM1* | cholinergic receptor, muscarinic 1 | 3376317 | 1,52 | 0,0101 |
| *CISD2* | CDGSH iron sulfur domain 2 | 2737840 | -1,56 | 0,0001 |
| *COG3* | component of oligomeric golgi complex 3 | 3488253 | -1,51 | 0,0001 |
| *COG6* | component of oligomeric golgi complex 6 | 3486383 | -1,70 | 0,0000 |
| *CRIP1* | cysteine-rich protein 1 (intestinal) | 3554851 | -1,76 | 0,0000 |
| *CSF1R* | colony stimulating factor 1 receptor | 2881187 | -1,62 | 0,0000 |
| *CTRB2 /// CTRB1* | chymotrypsinogen B2 // chymotrypsinogen B1 | 3699390 | 1,70 | 0,0001 |
| *CWF19L2* | CWF19-like 2, cell cycle control (S. pombe) | 3389745 | -1,52 | 0,0001 |
| *CXCL2* | chemokine (C-X-C motif) ligand 2 | 2773434 | 1,92 | 0,0000 |
| *CXorf21* | chromosome X open reading frame 21 | 4003895 | -1,63 | 0,0003 |
| *DCTN2* | dynactin 2 (p50) | 3458614 | -1,52 | 0,0006 |
| *DDX50* | DEAD (Asp-Glu-Ala-Asp) box polypeptide 50 | 3250019 | -1,60 | 0,0000 |
| *DHCR7* | 7-dehydrocholesterol reductase | 3380697 | -1,89 | 0,0001 |
| *DHDDS* | dehydrodolichyl diphosphate synthase | 2326496 | -1,65 | 0,0000 |
| *DIABLO* | diablo homolog (Drosophila) | 3475511 | -1,51 | 0,0004 |
| *DLST* | dihydrolipoamide S-succinyltransferase (E2 component of 2-oxo-glutarate complex) | 3544346 | -1,62 | 0,0004 |
| *DNAJC17 /// ZFYVE19* | DnaJ (Hsp40) homolog, subfamily C, member 17 // zinc finger, FYVE domain containing 19 | 3619650 | -1,59 | 0,0012 |
| *ELP2* | elongation protein 2 homolog (S. cerevisiae) | 3784727 | -1,59 | 0,0000 |
| *EMR1* | egf-like module containing, mucin-like, hormone receptor-like 1 | 3818596 | -1,61 | 0,0016 |
| *FADS1* | fatty acid desaturase 1 | 3375545 | -1,63 | 0,0000 |
| *FADS2* | fatty acid desaturase 2 | 3333247 | -1,62 | 0,0000 |
| *FAM188A* | family with sequence similarity 188, member A | 3279410 | -1,56 | 0,0004 |
| *FAM63B* | family with sequence similarity 63, member B | 3595846 | -1,54 | 0,0020 |
| *FAM64A* | family with sequence similarity 64, member A | 3707950 | 1,84 | 0,0011 |
| *FGFBP2* | fibroblast growth factor binding protein 2 | 2761837 | 1,60 | 0,0035 |
| *FITM2* | fat storage-inducing transmembrane protein 2 | 3906852 | -1,60 | 0,0018 |
| *FKBP9L /// FKBP9* | FK506 binding protein 9, 63 kDa | 2996246 | 1,76 | 0,0009 |
| *FN1* | fibronectin 1 | 2598261 | -2,06 | 0,0000 |
| *FOXL2* | forkhead box L2 | 2697652 | 1,53 | 0,0000 |
| *FSD1L /// RALGAPA1* | fibronectin type III and SPRY domain containing 1-like | 3183238 | -1,63 | 0,0046 |
| *GEMIN7* | gem (nuclear organelle) associated protein 7 | 3836044 | -1,50 | 0,0026 |
| *GPCPD1* | glycerophosphocholine phosphodiesterase GDE1 homolog (S. cerevisiae | 3896370 | -1,50 | 0,0001 |
| *GPR139* | G protein-coupled receptor 139 | 3683430 | 1,89 | 0,0000 |
| *GPR180* | G protein-coupled receptor 180 | 3496916 | -1,54 | 0,0004 |
| *HDGFL1* | hepatoma derived growth factor-like 1 | 2898096 | 1,56 | 0,0000 |
| *HIST1H1A* | histone cluster 1, H1a | 2946194 | 1,59 | 0,0005 |
| *HIST1H2AL* | histone cluster 1, H2al | 2900091 | 1,80 | 0,0004 |
| *HMGCR* | 3-hydroxy-3-methylglutaryl-CoA reductase | 2815965 | -1,54 | 0,0001 |
| *HMGCS1* | 3-hydroxy-3-methylglutaryl-CoA synthase 1 (soluble) | 2855501 | -2,10 | 0,0000 |
| *HNRNPA1L2 /// HNRNPA1P10 /// HNRNPA1* | heterogeneous nuclear ribonucleoprotein A1 // heterogeneous nuclear ribonucleoprotein A1 pseudogene 10// heterogeneous nuclear ribonucleoprotein A1-like 2 | 3416483 | -1,52 | 0,0036 |
| *IFT52* | intraflagellar transport 52 homolog (Chlamydomonas) | 3886179 | -1,51 | 0,0005 |
| *IFT74* | intraflagellar transport 74 homolog (Chlamydomonas) | 3165780 | -1,54 | 0,0073 |
| *IL16 /// STARD5* | StAR-related lipid transfer (START) domain containing 5 //interleukin 16 (lymphocyte chemoattractant factor) | 3635553 | -1,95 | 0,0000 |
| *IL18* | interleukin 18 (interferon-gamma-inducing factor) | 3391255 | -1,61 | 0,0000 |
| *IMPACT* | Impact homolog (mouse) | 3782166 | -1,67 | 0,0001 |
| *INGX* | inhibitor of growth family, X-linked, pseudogene | 4011951 | 1,54 | 0,0001 |
| *INO80C* | INO80 complex subunit C | 3804000 | -1,61 | 0,0000 |
| *ITGA3* | / integrin, alpha 3 (antigen CD49C, alpha 3 subunit of VLA-3 receptor) | 3726154 | -1,54 | 0,0002 |
| *KAT5* | K(lysine) acetyltransferase 5 | 3335517 | -1,50 | 0,0023 |
| *KCNE3* | potassium voltage-gated channel, Isk-related family, member 3 | 3381965 | -1,58 | 0,0020 |
| *KCNE4* | potassium voltage-gated channel, Isk-related family, member 4 | 2529627 | 1,75 | 0,0077 |
| *KCNK3* | potassium channel, subfamily K, member 3 | 2473936 | 1,68 | 0,0000 |
| *KDM4C* | lysine (K)-specific demethylase 4C | 3161566 | -1,75 | 0,0001 |
| *KIAA0528* | KIAA0528 | 3447129 | -1,50 | 0,0007 |
| *KIAA1826* | KIAA1826 | 3389529 | -1,64 | 0,0013 |
| *KLHDC2* | kelch domain containing 2 | 3534923 | -1,67 | 0,0000 |
| *KRTAP5-5 /// KRTAP5-7 /// KRTAP5-8 /// KRTAP5-3* | keratin associated protein 5-3 // keratin associated protein 5-8 //keratin associated protein 5-7 // keratin associated protein 5-5 //keratin associated protein 5-3 | 3358906 | 3,01 | 0,0000 |
| *KRTAP6-3* | keratin associated protein 6-3 | 3917582 | 1,88 | 0,0024 |
| *LCE3A* | late cornified envelope 3A | 2435745 | 1,54 | 0,0006 |
| *LHFPL3* | lipoma HMGIC fusion partner-like 3 | 3017354 | 1,67 | 0,0000 |
| *LHFPL4* | lipoma HMGIC fusion partner-like 4 | 2662297 | 1,54 | 0,0023 |
| *LOC100101266 /// HAVCR1* | hepatitis A virus cellular receptor 1// hepatitis A virus cellular receptor 1 pseudogene | 2883317 | -1,69 | 0,0055 |
| *LOC100129518 /// ACAT2* | acetyl-CoA acetyltransferase 2 | 2934131 | -1,56 | 0,0000 |
| *LOC100132273 /// C22orf32* | chromosome 22 open reading frame 32 | 3947310 | -1,60 | 0,0006 |
| *LOC100132481 /// CHCHD6* | coiled-coil-helix-coiled-coil-helix domain containing 6 | 2640507 | -1,54 | 0,0095 |
| *LOC100507488 /// FN1* | NMfibronectin 1 | 2526806 | -1,64 | 0,0005 |
| *LOC399815 /// C10orf88* | chromosome 10 open reading frame 88 | 3310725 | -1,55 | 0,0030 |
| *LOC80054* | hypothetical LOC80054 | 3829300 | 2,84 | 0,0002 |
| *LRRC37A3 /// LRRC37A2 /// LRRC37A4 /// ARL17A /// LRRC37A* | leucine rich repeat containing 37, member A3 //ADP-ribosylation factor-like 17A // leucine rich repeat containing 37, member A2 // LRRC37A4 // leucine rich repeat containing 37, member A4 (pseudogene) | 3767169 | -1,87 | 0,0012 |
| *LRRFIP1* | leucine rich repeat (in FLII) interacting protein 1 | 2534456 | -1,84 | 0,0003 |
| *MASTL* | microtubule associated serine/threonine kinase-like | 3240012 | -1,66 | 0,0001 |
| *MDP1* | magnesium-dependent phosphatase 1 | 3557968 | -1,72 | 0,0012 |
| *MEGF8* | multiple EGF-like-domains 8 | 3834837 | -1,55 | 0,0089 |
| *MMP2* | matrix metallopeptidase 2 (gelatinase A, 72kDa gelatinase, 72kDa type IV collagenase) | 3661684 | -1,52 | 0,0004 |
| *MMP3* | matrix metallopeptidase 3 (stromelysin 1, progelatinase) | 3388830 | 1,66 | 0,0000 |
| *MNDA* | myeloid cell nuclear differentiation antigen | 2362333 | -1,54 | 0,0075 |
| *MOGAT2* | monoacylglycerol O-acyltransferase 2 | 3340640 | 1,69 | 0,0004 |
| *MS4A4A* | membrane-spanning 4-domains, subfamily A, member 4 | 3332298 | -1,56 | 0,0002 |
| *MT1G* | metallothionein 1G | 3692999 | 1,81 | 0,0026 |
| *MT3* | metallothionein 3 | 3662093 | 1,62 | 0,0001 |
| *MUCL1* | mucin-like 1 | 3416702 | 1,54 | 0,0082 |
| *MYCN* | v-myc myelocytomatosis viral related oncogene, neuroblastoma derived (avian) | 2470805 | 1,72 | 0,0017 |
| *MYST4* | MYST histone acetyltransferase (monocytic leukemia) 4 | 3252382 | -1,57 | 0,0000 |
| *NACAP1* | nascent-polypeptide-associated complex alpha polypeptide pseudogene 1 | 3109600 | 1,92 | 0,0000 |
| *NAPB* | N-ethylmaleimide-sensitive factor attachment protein, beta | 3901191 | -1,52 | 0,0013 |
| *NAT10* | N-acetyltransferase 10 (GCN5-related) | 3326252 | -1,52 | 0,0001 |
| *NCF4* | neutrophil cytosolic factor 4, 40kDa | 3944543 | -1,52 | 0,0000 |
| *NCR1* | natural cytotoxicity triggering receptor 1 | 3841881 | -1,51 | 0,0002 |
| *NCRNA00257* | non-protein coding RNA 257 | 3932261 | 1,93 | 0,0087 |
| *NEIL1 /// MAN2C1* | nei endonuclease VIII-like 1 (E. coli) // mannosidase, alpha, class 2C, member 1 | 3602299 | 1,57 | 0,0001 |
| *NFX1* | Nuclear transcription factor, X-box binding 1 | 3166880 | -1,59 | 0,0001 |
| *NKPD1 /// TRAPPC6A* | trafficking protein particle complex 6A | 3865223 | -1,57 | 0,0036 |
| *NOB1* | RPN12 binding protein 1 homolog (S. cerevisiae) | 3696697 | -1,69 | 0,0013 |
| *NXF2 /// NXF2B* | nuclear RNA export factor 2 | 3985034 | -2,09 | 0,0052 |
| *OR4A5* | olfactory receptor, family 4, subfamily A, member 5 | 3373122 | 1,62 | 0,0001 |
| *OR52M1* | olfactory receptor, family 52, subfamily M, member 1 | 3318141 | 1,68 | 0,0004 |
| *OSGEP* | O-sialoglycoprotein endopeptidase | 3555461 | -1,62 | 0,0000 |
| *PAN3* | PAN3 poly(A) specific ribonuclease subunit homolog (S. cerevisiae) | 3483159 | -1,51 | 0,0018 |
| *PBLD* | phenazine biosynthesis-like protein domain containing | 3292590 | -1,76 | 0,0009 |
| *PCID2* | PCI domain containing 2 | 3526378 | -1,55 | 0,0007 |
| *PCOLCE2* | procollagen C-endopeptidase enhancer 2 | 2698996 | -1,74 | 0,0000 |
| *PCSK1N* | proprotein convertase subtilisin/kexin type 1 inhibitor | 4007550 | 1,53 | 0,0003 |
| *PDXP /// SH3BP1* | pyridoxal (pyridoxine, vitamin B6) phosphatase //SH3-domain binding protein 1 | 3944873 | 1,52 | 0,0001 |
| *PFN4* | profilin family, member 4 | 2544219 | 1,53 | 0,0026 |
| *PIH1D1* | PIH1 domain containing 1 | 3867865 | -1,53 | 0,0001 |
| *PIR* | pirin (iron-binding nuclear protein) | 4000560 | -1,72 | 0,0004 |
| *PLEKHA1* | pleckstrin homology domain containing, family A (phosphoinositide binding specific) member 1 | 3268274 | -1,65 | 0,0005 |
| *PLEKHF2* | pleckstrin homology domain containing, family F (with FYVE domain) member 2 | 3107828 | -1,57 | 0,0026 |
| *POLI* | polymerase (DNA directed) iota | 3788833 | -1,58 | 0,0038 |
| *PROKR1* | prokineticin receptor 1 | 2486901 | 1,58 | 0,0014 |
| *PSD3* | / pleckstrin and Sec7 domain containing 3 | 3126368 | -2,12 | 0,0013 |
| *PSMA2 /// C7orf25* | chromosome 7 open reading frame 2 | 3047953 | -1,62 | 0,0040 |
| *PSME2* | proteasome (prosome, macropain) activator subunit 2 (PA28 beta) | 3557811 | 2,90 | 0,0000 |
| *RBM43* | RNA binding motif protein 43 | 2580943 | -2,03 | 0,0004 |
| *RGS2* | regulator of G-protein signaling 2, 24kDa | 2372858 | -1,81 | 0,0001 |
| *RHOB* | ras homolog gene family, member B | 2471978 | 1,57 | 0,0000 |
| *RNF185* | ring finger protein 185 | 3957738 | -2,01 | 0,0055 |
| *ROGDI* | rogdi homolog (Drosophila) | 3678369 | -1,52 | 0,0002 |
| *RPL23AP64* | NR_003040 // RPL23AP64 // ribosomal protein L23a pseudogene 64 // 11q23.3 // 649946 /// BC017930 // RPL23AP64 // ribosomal protein L23a pseudogene 64 // 11q23.3 // 649946 | 3394057 | 2,68 | 0,0006 |
| *RPLP0* | ribosomal protein, large, P0 | 3474344 | 2,17 | 0,0000 |
| *RPP38* | ribonuclease P/MRP 38kDa subunit | 3236538 | -1,67 | 0,0037 |
| *RPS11* | ribosomal protein S11 | 3439256 | -1,62 | 0,0037 |
| *RSF1* | NM_ remodeling and spacing factor 1 | 3382972 | -1,60 | 0,0001 |
| *S100B* | S100 calcium binding protein B | 3935486 | -1,57 | 0,0005 |
| *S1PR2* | sphingosine-1-phosphate receptor 2 | 3850166 | 1,64 | 0,0056 |
| *SC4MOL* | sterol-C4-methyl oxidase-like | 2750594 | -1,95 | 0,0000 |
| *SC5DL* | sterol-C5-desaturase | 3352904 | -1,52 | 0,0006 |
| *SCFD1* | sec1 family domain containing 1 | 3531032 | -1,51 | 0,0010 |
| *SCFD2* | / sec1 family domain containing 2 | 2769182 | -1,66 | 0,0016 |
| *SEC23A* | Sec23 homolog A (S. cerevisiae) | 3561952 | -1,54 | 0,0008 |
| *SEPT11* | septin 11 | 2732273 | 1,82 | 0,0000 |
| *SH3BGRL /// HMGN5* | high-mobility group nucleosome binding domain 5 | 4013828 | -1,54 | 0,0061 |
| *SIGLEC11* | sialic acid binding Ig-like lectin 11 | 3868257 | 1,51 | 0,0001 |
| *SIRT2* | sirtuin 2 | 3861689 | -1,54 | 0,0019 |
| *SLC25A16* | solute carrier family 25 (mitochondrial carrier; Graves disease autoantigen), member 16 | 3292735 | -1,58 | 0,0001 |
| *SLC29A1* | solute carrier family 29 (nucleoside transporters), member 1 | 2908423 | -1,53 | 0,0000 |
| *SLC35E4 /// DUSP18* | dual specificity phosphatase 18 //solute carrier family 35, member E4 | 3957486 | -1,60 | 0,0002 |
| *SLC39A8* | solute carrier family 39 (zinc transporter), member 8 | 2779823 | -1,61 | 0,0003 |
| *SLFN5* | schlafen family member 5 | 3718555 | -1,52 | 0,0000 |
| *SNORA17 /// SNORA43 /// SNHG7* | small nucleolar RNA host gene 7 (non-protein coding) // small nucleolar RNA, H/ACA box 17 // small nucleolar RNA, H/ACA box 43 | 3230332 | 1,76 | 0,0007 |
| *SNORA2A /// SNORA2B /// SNORA34 /// C12orf41* | chromosome 12 open reading frame 41 | 3453177 | -1,84 | 0,0000 |
| *SNUPN* | snurportin 1 | 3633522 | -1,62 | 0,0002 |
| *STARD4* | StAR-related lipid transfer (START) domain containing 4 | 2870828 | -1,92 | 0,0000 |
| *STAT2* | signal transducer and activator of transcription 2, 113kDa | 3457752 | -1,90 | 0,0000 |
| *STC1* | stanniocalcin 1 | 3128046 | 1,71 | 0,0000 |
| *SUMO1P1* | SUMO1 pseudogene 1 | 3910347 | -1,58 | 0,0025 |
| *SUMO1P3 /// SUMO1* | SMT3 suppressor of mif two 3 homolog 1 (S. cerevisiae) // SMT3 suppressor of mif two 3 homolog 1 (S. cerevisiae) | 2595252 | 1,73 | 0,0003 |
| *SUMO1P3 /// SUMO1* | SUMO1 pseudogene 3 // SMT3 suppressor of mif two 3 homolog 1 (S. cerevisiae) | 2363074 | -2,63 | 0,0071 |
| *SYNGR2* | synaptogyrin 2 | 3736232 | -1,55 | 0,0007 |
| *TAGLN3* | transgelin 3 | 2635998 | 1,51 | 0,0007 |
| *TCEAL3 /// TCEAL6* | transcription elongation factor A (SII)-like 3// transcription elongation factor A (SII)-like 6 | 3985644 | 1,65 | 0,0002 |
| *TCEAL7* | transcription elongation factor A (SII)-like 7 | 3985511 | 1,60 | 0,0018 |
| *TCEANC* | transcription elongation factor A (SII) N-terminal and central domain containing | 3969396 | -1,73 | 0,0013 |
| *TCF25* | transcription factor 25 (basic helix-loop-helix) | 3674434 | -1,53 | 0,0000 |
| *TDP1* | tyrosyl-DNA phosphodiesterase 1 | 3548152 | -1,51 | 0,0004 |
| *TEX9* | testis expressed 9 | 3594986 | -1,53 | 0,0035 |
| *TGDS* | TDP-glucose 4,6-dehydratase | 3520989 | -1,61 | 0,0001 |
| *TIAM2 /// TFB1M* | transcription factor B1, mitochondrial | 2980812 | -1,54 | 0,0020 |
| *TIGD6 /// SLC26A2* | solute carrier family 26 (sulfate transporter), member 2 | 2835300 | -1,65 | 0,0040 |
| *TKTL2* | transketolase-like 2 | 2792161 | 1,69 | 0,0000 |
| *TMEM145* | transmembrane protein 145 | 3834744 | 1,68 | 0,0002 |
| *TMEM208* | transmembrane protein 208 | 3665357 | -1,52 | 0,0000 |
| *TMEM45A* | transmembrane protein 45A | 2633691 | -1,67 | 0,0031 |
| *TPP1* | tripeptidyl peptidase I | 3361041 | -1,55 | 0,0000 |
| *TRAPPC4* | trafficking protein particle complex 4 | 3351775 | -1,50 | 0,0000 |
| *TRIM21* | tripartite motif-containing 21 | 3360142 | -1,53 | 0,0039 |
| *TRUB1* | TruB pseudouridine (psi) synthase homolog 1 (E. coli) | 3265494 | 1,66 | 0,0000 |
| *TTC39C* | tetratricopeptide repeat domain 39C | 3781980 | -1,58 | 0,0004 |
| *TUBA3C /// TUBA3D /// TUBA3E* | tubulin, alpha 3e// tubulin, alpha 3d// tubulin, alpha 3c | 2575949 | 2,64 | 0,0081 |
| *TUBB2B* | tubulin, beta 2B | 2939232 | 1,69 | 0,0023 |
| *TXNIP* | thioredoxin interacting protein | 2356115 | 1,59 | 0,0002 |
| *UFSP2* | UFM1-specific peptidase 2 | 2796875 | -1,60 | 0,0008 |
| *VIPAR* | VPS33B interacting protein, apical-basolateral polarity regulator | 3573078 | -1,55 | 0,0000 |
| *WNT7B* | wingless-type MMTV integration site family, member 7B | 4053903 | 1,92 | 0,0011 |
| *ZBED5* | zinc finger, BED-type containing 5 | 3362934 | -1,53 | 0,0083 |
| *ZBTB41* | zinc finger and BTB domain containing 41 | 2449619 | -1,51 | 0,0010 |
| *ZFC3H1* | zinc finger, C3H1-type containing | 3462094 | -1,50 | 0,0005 |
| *ZNF25* | zinc finger protein 25 | 3285614 | -1,54 | 0,0081 |
| *ZNF263 /// TIGD7* | tigger transposable element derived 7 //zinc finger protein 263 | 3677538 | -1,58 | 0,0015 |
| *ZNF460* | zinc finger protein 460 | 3843156 | -1,62 | 0,0005 |
| *ZNF841 /// ZNF836 /// ZNF432* | zinc finger protein 841 // zinc finger protein 432 // zinc finger protein 836 | 3869396 | -1,98 | 0,0002 |
